# Vulnerability of the entorhinal cortex II to neurodegeneration in Alzheimer’s disease

**DOI:** 10.1093/braincomms/fcaf091

**Published:** 2025-02-26

**Authors:** Aldelmo Emmanuel Reyes-Pablo, Nabil Itzi Luna-Viramontes, José Francisco Montiel-Sosa, Miguel Ángel Ontiveros-Torres, Linda Garcés-Ramírez, Fidel de la Cruz-López, Ricardo Apátiga-Pérez, Ignacio Villanueva-Fierro, Mario Hernandes-Alejandro, Blanca Estela Jaramillo-Loranca, Genaro Vargas-Hernández, Mar Pacheco-Herrero, José Luna-Muñoz

**Affiliations:** National Dementia BioBank, AMPAEYDEN A.C., and Federación Mexicana de Alzheimer, Estado de México CP 54743, México; Escuela Nacional de Ciencias Biológicas, Depto. de Fisiología, Instituto Politécnico Nacional, Ciudad de México CP 07700, México; National Dementia BioBank, AMPAEYDEN A.C., and Federación Mexicana de Alzheimer, Estado de México CP 54743, México; Escuela Nacional de Ciencias Biológicas, Depto. de Fisiología, Instituto Politécnico Nacional, Ciudad de México CP 07700, México; Departamento de Ciencias Biológicas, Facultad de Estudios Superiores, UNAM, Estado de México CP 54714, México; School of Engineering and Science, Tecnologico de Monterrey, Toluca CP 64849, México; Escuela Nacional de Ciencias Biológicas, Depto. de Fisiología, Instituto Politécnico Nacional, Ciudad de México CP 07700, México; Escuela Nacional de Ciencias Biológicas, Depto. de Fisiología, Instituto Politécnico Nacional, Ciudad de México CP 07700, México; National Dementia BioBank, AMPAEYDEN A.C., and Federación Mexicana de Alzheimer, Estado de México CP 54743, México; Escuela Nacional de Ciencias Biológicas, Depto. de Fisiología, Instituto Politécnico Nacional, Ciudad de México CP 07700, México; Instituto Politécnico Nacional, CIIDIR, Unidad Durango, Becario COFAA, Durango CP 34220, México; Departamento de Bioingeniería, Unidad Profesional Interdisciplinaria de Biotecnología del Instituto Politécnico Nacional, Ciudad de México CP 07340, México; Dirección de Investigación, Innovación y Posgrado, Universidad Politécnica de Pachuca, Zempoala, Zempoala, Hidalgo CP 43830, México; Programa Educativo Posgrado en Biotecnología, Universidad Politécnica de Pachuca Zempoala, Zempoala, Hidalgo CP 43830, México; Neuroscience Research Laboratory, Faculty of Health Sciences, Pontificia Universidad Católica Madre y Maestra, Santiago de los Caballeros CP 51000, Dominican Republic; National Dementia BioBank, AMPAEYDEN A.C., and Federación Mexicana de Alzheimer, Estado de México CP 54743, México; Dirección de Investigación, Innovación y Posgrado, Universidad Politécnica de Pachuca, Zempoala, Zempoala, Hidalgo CP 43830, México; Banco Nacional de Cerebros-UNPHU, Universidad Nacional Pedro Henríquez Ureña, Santo Domingo CP 10603, República Dominicana

**Keywords:** entorhinal cortex, neuronal vulnerability, Alzheimer's disease, regional conformational change, phosphorylated tau protein

## Abstract

Alzheimer's disease is characterized by progressive memory loss and deterioration of cognitive functions. The presence of neurofibrillary tangles in the hippocampal areas (perforant pathway) correlates with cognitive impairment. Pathological processing of tau protein is characterized by post-translational changes such as hyperphosphorylation and truncation, which favour conformational changes within tau. These conformational changes can be regional (dependent on phosphorylation) or structural (depending on regional conformational changes and truncation). Through immunohistochemical and immunofluorescence staining in hippocampus Alzheimer disease brains and quantification in tissue stained with TG3 antibody and analysed by confocal microscopy, we have been able to demonstrate that TG3 correlates with cognitive impairment. In the process of tangle evolution, TG3 is present in pre-tangle. This epitope of the TG3 antibody was very stable to proteolytic processing by caspase-3; truncation is evidenced by the TauC-3 antibody. The entorhinal cortex showed high sensitivity to neurodegeneration and pathological tau processing.

## Teaser

In Alzheimer's disease, pathological tau processing is characterized by post-translational changes such as hyperphosphorylation and truncation. TG3 correlates with cognitive impairment and is present in pre-Neurofibrillary tangle. This epitope was very stable to proteolytic processing by caspases. The entorhinal cortex showed high sensitivity to pathological tau processing.

## Graphical Abstract

**Graphical Abstract:**
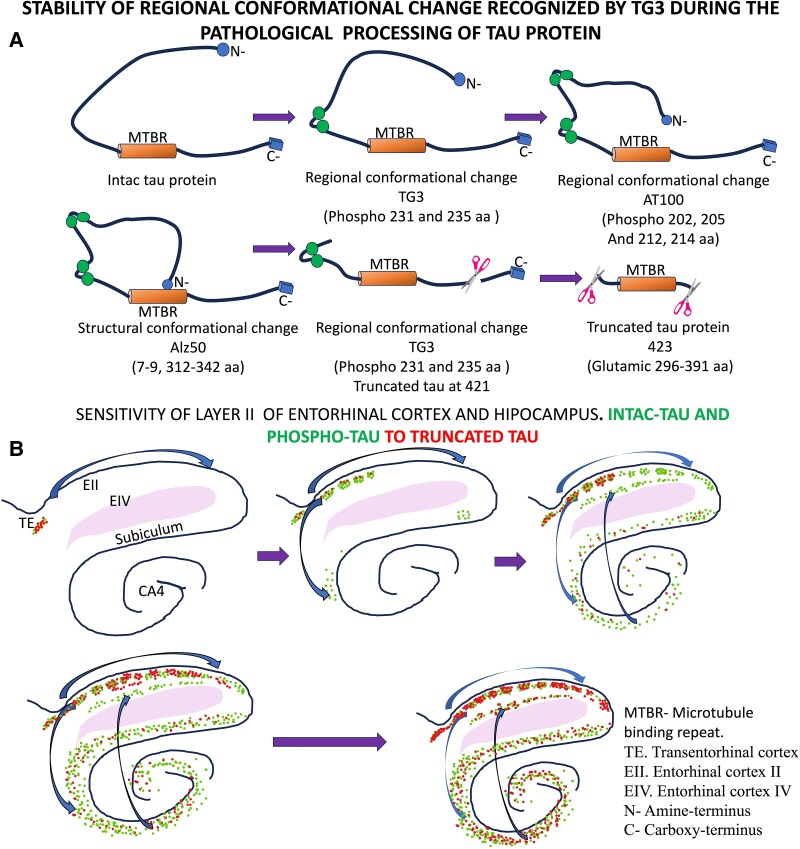

## Introduction

Alzheimer's disease (AD) is a neurodegenerative disease with the highest incidence among dementias (65–75%).^1^ Clinically, it has been characterized by progressive loss of short-term memory and alterations in judgment and behaviour.^2^ The lesions that characterize AD brains are neuritic plaques, lesions constituted by extracellular deposits of amyloid beta (Aβ), which favour an inflammatory environment sustained by glial and microglial cells.^3-5^ The other lesion observed is the presence of neurofibrillary tangles (NFTs). Cognitive impairment in individuals has been associated with the presence of NFTs in the hippocampus.^3,6-8^ NFTs are formed from a massive accumulation of paired helical filaments (PHFs) in the neuronal soma. These PHFs are mainly constituted by the tau protein, which undergoes post-translational modifications such as hyperphosphorylation and truncation. Both events have been associated with the genesis and polymerization of PHFs^9-15^ Within the aggregation processing of tau protein in the neuronal soma, it has been characterized by a diffuse granular accumulation that culminates in the extracellular NFT.^15-18^ Among the markers so far used for the evaluation and pathological marking of AD, no specific marker has been shown to differentiate AD from other tauopathies, in which the tau protein is also modified, showing hyperphosphorylation and truncation at Asp421 (recognized by the marker TauC-3).^19^ Previous observation our laboratory showed a sequence of specific events stereotyped in preNFT before tau is assembled into filaments. The sequence of the markers is as follows: pT231, TG3, AT8, AT100 and Alz50. After tau protein is phosphorylated at pT231, the presence of a truncation at Asp421 is observed.^15^ The pathological processing of tau associated with the different stages of phosphorylation is difficult to demonstrate in well-formed NFTs because in PHFs all the modified tau species are aggregated, showing phosphorylation events, truncation, regional and structural conformational changes, as well as intact tau molecules in their N- and C-terminal portions.^18^ Our objective was to evaluate the aggregation and processing pattern of the TG3 antibody, a marker that recognizes a regional phosphorylation-dependent conformational change in amino acids T231 and S235, as well as its implication with the degree of cognitive impairment of the Alzheimer's disease patient. In our study, we demonstrated the presence of immunoreactive NFTs in the hippocampus of patients with different stages of cognitive impairment. When comparing the expression of lesions with the TG3 antibody against AT100 and Alz50, we found that the affinity of TG3 is more stable compared to other markers associated with its N-terminal and C-terminal portions. TG3 also showed a close relationship with phosphorylation markers associated with its C-terminal portion, such as phosphorylation at amino acid 396 (evidenced by the pS396 antibody), 396 and 404 (AD2 marker). In our analysis, we demonstrated that these markers are mainly expressed when the tau protein is assembled in filaments, and its immunoreactivity can persist until the formation of a transitional NFT towards the extracellular NFT. Likewise, the number of NFTs immunoreactive to the TG3 marker showed a correlation with the degree of cognitive impairment. Our results suggest that TG3 could be a specific marker to differentiate pathological processing in AD, as well as that this marker correlates with cognitive impairment in the evolution of this disease.

## Material and methods

Brain tissue from subjects with AD and healthy subjects was used from two different collections: (i) 10% formalin-fixed tissue from the National Dementia BioBank. AMPAEYDEN A.C., corresponding to Mexican subjects of both sexes, advanced age and preserved at 4°C. Table 1; and (ii) paraffin-embedded brain tissue from the Cambridge University Brain Bank, known as CPLL (Cambridge Program for Longer Life), classified by neuropathology (Braak stages) and clinical severity (CAMDEX-Cambridge Examination for Mental Disorders of the Elderly).^20^ See Tables 2 and 3.

**Table 1:** Brain BND

Case number	Age	Sex	Diagnostic
1	71	Male	Alzheimer’s disease
2	66	Female	Alzheimer’s disease
3	82	Female	Alzheimer’s disease
4	73	Male	Alzheimer’s disease
5	67	Female	Alzheimer’s disease

**Table 2:** Demographic information of Cambridge project for long life cases

CPLL	Braak stage	Sex	Age	APOE	CAMDEX
L22	1	M	93	E2/E2	0
L35	1	F	86	E2/E3	1
L62	1	M	87	E2/E4	0
L26	2	F	88	E3/E3	3
L39	2	F	86	E3/E3	2
L3	2	M	81	E3/E4	0
L13	2	M	89	E3/E3	0
L16	2	M	85	E3/E3	0
L50	2	F	86	E3/E3	1
L66	2	M	87	E4/E4	0
L10	3	F	88	E3/E4	3
L40	3	F	98	E3/E3	2
L17	3	F	84	E3/E3	0
L20	3	F	90	E3/E3	0
L21	3	M	89	E3/E4	1
L27	3	F	86	E3/E3	0
L28	3	F	88	E3/E4	1
L38	3	F	91	E3/E3	0
L43	3	M	91	E2/E3	1
L47	3	F	90	E3/E3	0
L48	3	F	86	E3/E4	0
L49	3	F	96	E3/E3	0
L54	3	M	89	E3/E3	1
L23	4	F	89	E3/E3	3
L57	4	F	89	E3/E3	4
L60	4	M	93	E3/E3	2
L18	4	M	85	E3/E4	1
L59	4	F	82	E2/E3	0
L61	4	F	89	E3/E3	0
L69	4	F	92	E3/E3	0
L7	5	F	89	E3/E4	4
L25	5	F	86	E3/E4	4
L30	5	M	89	E3/E4	3
L42	5	F	86	E3/E3	2
L45	5	F	91	E3/E4	3
L55	5	M	88	E3/E4	2
L56	5	M	89	E3/E4	3
L11	5	F	89	E2/E3	1
L51	5	F	91	E3/E4	1
L24	6	F	81	E4/E4	3

AD, Alzheimer disease; ApoE, apolipoprotein; CAMDEX, Cambridge Examination for Mental Disorder of the early; CPLL, Cambrigge Proyec for long life.

**Table 3:** Clinical dementia severity scale, used by the university of Cambridge brain bank in the study of CPLL* cases

Severity stage	Clinical expression of dementia
0	Absence of expression (normal subject)
1	Minimal: preclinical stage of manifestation
2	Average: initial manifestation
3	Moderate: well established dementia
4	Severe dementia

### Statistical analysis

Kruskal–Wallis One Way Analysis of Variance on Ranks was performed for Braak’ stage comparation versus NFTs. Two-way repeated measures ANOVA (with one factor repetition) was applied to tangle counts. We compared these between AD and control case groups. Two-way analysis of variance was performed for tangle counts when comparing Braak's stage and area; to compare the NFTs versus the area in AD and control groups either for TG3 or AT8; also, was run for tangle counts when comparing ApoE, AD and control groups. Paired sample *t*-test was performed to compare the number of NFTs by mm^2^ labelled with AT8 versus TG3 by area.

Nonparametric Spearman correlation coefficients were calculated to correlate clinic-pathological parameters with tangle counts. Statistical analysis was conducted using a sigma plot for the Windows software package.

### Immunocytochemistry

Immunocytochemistry studies were performed using the TG3 antibody. Tissue sections 7–10 μm thick from paraffin-embedded brains of CPLL cases were used for this study.

For the neuropathological characterization of the lesions recognized by the TG3 antibody, tissue sections of 5 μm were obtained.

First, sections were deparaffinized with xylol solution (10 min at room temperature), ethanol (98–50% solution train for 5 min) and finally kept in PBS (30 min, pH 7.4), for subsequent immunoperoxidase processing. The endogenous peroxidase was then neutralized with a 0.03% hydrogen peroxide solution (Sigma Co) for 10 min at room temperature. Additionally, the sections were blocked with albumin and incubated with the primary antibody in PBS-Triton (PBS 0.2% Sigma Co.) overnight at 4°C diluted in PBS-T.

The sections were then incubated with the peroxidase-coupled secondary antibody (HPR) for 1 h at room temperature. The peroxidase reaction was performed in PBS-T with diamino benzidine (DAB, Sigma Co) and 0.003% hydrogen peroxide for 8 min. Sections were counterstained with Cresyl violet and mounted in DPX permanent resin (Sigma Co).^21^

### Quantimetry and morphometry

A morphometric analysis was performed involving the counting of NFTs in the following areas: transentorhinal region, entorhinal cortex Layers II (ERC II) and IV (ERC IV), CA1/subiculum area. This morphometric quantification in the hippocampal formation was performed by determining the number of NFTs from three different random fields for each of the areas, and a summation was made to obtain the number of NFTs/mm^2^.

Observations were made by brightfield microscopy using 4 × and 20 × objectives, as well as 40 × and 100 × with AN of 0.7 and 1.4 respectively, for more precise observations.

The number of NFTs was correlated with respect to various neuropathological and clinical parameters, such as Braak stage and clinical severity. The number of structures was compared between cases with AD and normal control subjects.^8^

### Immunofluorescence

For the analysis of the neuropathological processing of tau protein and its association with the conformational change evidenced by the TG3 antibody, brain slices from the National Dementia BioBank AMPAEYDEN A.C. were used. Sections were blocked with IgG-free albumin (Sigma, Co) in 1% PBS/triton and then incubated with the primary antibodies overnight at 4°C (see Table 4). The specific secondary antibody(s) coupled to the different fluorochromes (FITC, TRITC or CY5) were incubated for 1 h at room temperature. Some sections were counterstained for 10 min with thiazine red dye (0.0002% in water) at room temperature in the dark.^35^ The immunoreactivity and colocalization patterns were evaluated by confocal microscopy.

**Table 4:** Antibodies and recognition sites

Antibody	Epitope (amino acid residue numbers)	Species and isotype	Reference, source
Alz-50	Tau 5–15, 312–322. Structural conformational change	Mo IgM	Carmel et al.^22^; Jicha et al.^23^
TG-3	Tau phospho-Thr231. Ser 235 Regional conformational change	Mo IgM	Jicha et al.^24^
pT231	Tau phospho-Thr231	Rb IgG	Thermo Fisher
AT100	Tau phospho-Ser202, Thr205, Thr212, Ser214. Regional conformational change	Mo IgG	Zheng-Fischhofer et al.^25^ Thermo Fisher
AD2	Tau phospho-Ser396, Ser404	Mo IgG	Buee-Scherrer et al.^26^
pSer396	Tau phospho-Ser396	Rb IgG	Thermo Fisher
pSer400	Tau phospho-Ser400	Rb IgG	Song et al.^27^ Thermo Fisher
pSer422	Tau phospho-Ser422	Rb IgG	Thermo Fisher
pSer199	Tau phospho-Ser199	Mo IgG	LoPresti et al.^28^
Tau-12	Tau dephosphorylated 195, 198, 199 and 202	Mo IgG	Binder et al.^29^
Tau-7	Tau C-terminus	Mo IgG	Horowitz et al.^30^
T46	Tau 404–441	Mo IgG	Merrick et al.^31^
M19			
TauC-3	Truncated tau at Asp421	Mo IgG	Sato et al.^32^
423	Truncated tau at Glu391	Mo IgG	Novak^33^
Thiazine Red (TR)	Fluorescent red dye (570 nm), tau and amyloid fibrillar with the beta pleated conformation.		Resch et al.^34^

### Confocal microscopy

Double and triple immunolabelled sections were mounted in anti-quenching media Vectashield (Vector Labs, Burlingame) and viewed through a confocal laser scanning microscope (TCP-SP8, Leica, Heidel-berg) using 20 × dry and 100 × oil-immersion plan Apochromatic objectives (NA 1.4). Ten to fifteen sequential single sections were obtained at 0.8–1.0 μm intervals and sequentially scanned in all channels throughout the *z*-axis of the sample. The resulting stack of images was projected and analysed onto the 2D plane using a pseudocolour display of green (FITC), red (TRITC) and blue (CY5). Fluorochromes in double- and triple-labelled samples were excited at 488 nm (for FITC), 540 nm (for TRITC) and 650 nm (for CY5). The autofluorescence of lipofuscin granules in AD brain tissue was observed in the red channel. In some tissues, the lipofuscin was blocked with Sudan black. The images were analysed using Leica SP8 software.

## Results

### Aggregation pattern of tau protein in neuronal soma in AD cases

**Figure 1:**
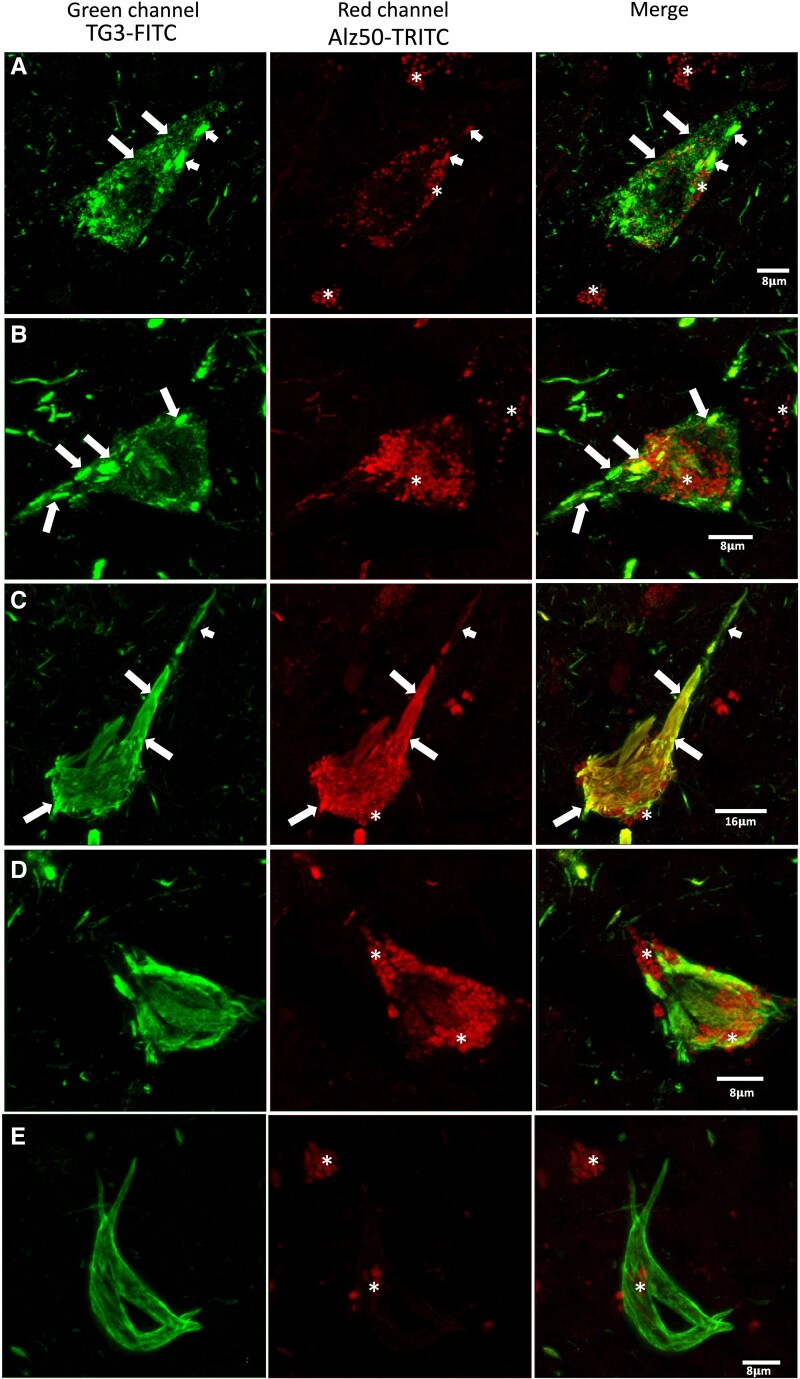
**Pattern of immunoreactivity of TG3 and Alz50 in the neuronal soma in AD.** (**A)** Diffuse granular staining immunoreactive to TG3. Small dense bundles of tau colocalize with Alz50. (**B)** Small NFTs in which TG3 and Alz50 colocalize (arrows). (**C)** Intracellular NFT where both markers colocalize abundantly (large arrows). In the apical dendrite, TG3 antibody immunoreactivity predominates (small arrow). (**D)** Intracellular NFT where Alz50 antibody immunoreactivity tends to be lost and TG3 prevails. (**E)** Transitional NFT evidenced by TG3 antibody.

By double immunostaining, we have been able to observe a sequence of tau protein aggregation in the neuronal soma. The first aggregation of tau is characterized by diffuse granular staining that is predominantly TG3 positive (Fig. 1A, large arrows). In the dense aggregates, colocalization of both TG3 and Alz50 markers is observed (Fig. 1A, small arrow). Dystrophic neurites positive for both antibodies are observed in the periphery, although mostly green (TG3) predominates. Lipofuscin is autofluorescent, observed with a coarse granular (* red channel). In the next stage of tau protein aggregation, dense bundles are observed within the neuronal soma (beed-like structures; Fig. 1B, arrows). These structures are scattered in the neuronal soma and the basal and apical dendrites (Fig. 1C, large arrows). Some NFT where recognized by both markers (TG3-Alz50) colocalize abundantly in most of the structure, whereas in the apical neurite (Fig. 1C, small arrow) TG3 antibody immunoreactivity is predominantly observed. Dystrophic neurites immunoreactive to TG3 are observed in the vicinity. In Fig. 1D, a NFT is observed that is characterized by a predominant expression of the TG3 antibody (arrow), colocalizing in some areas with the markers (TG3-Alz50). In neurites in the vicinity, colocalization of both markers is abundantly observed, and in another population of neurons TG3 is preferentially present. In another neuronal population, an exclusively immunoreactive staining to TG3 antibody was observed, this NFT did not show affinity for the Alz50 antibody (Fig. 1E).

### Triple immunostaining TG3-TauC-3-S199

**Figure 2:**
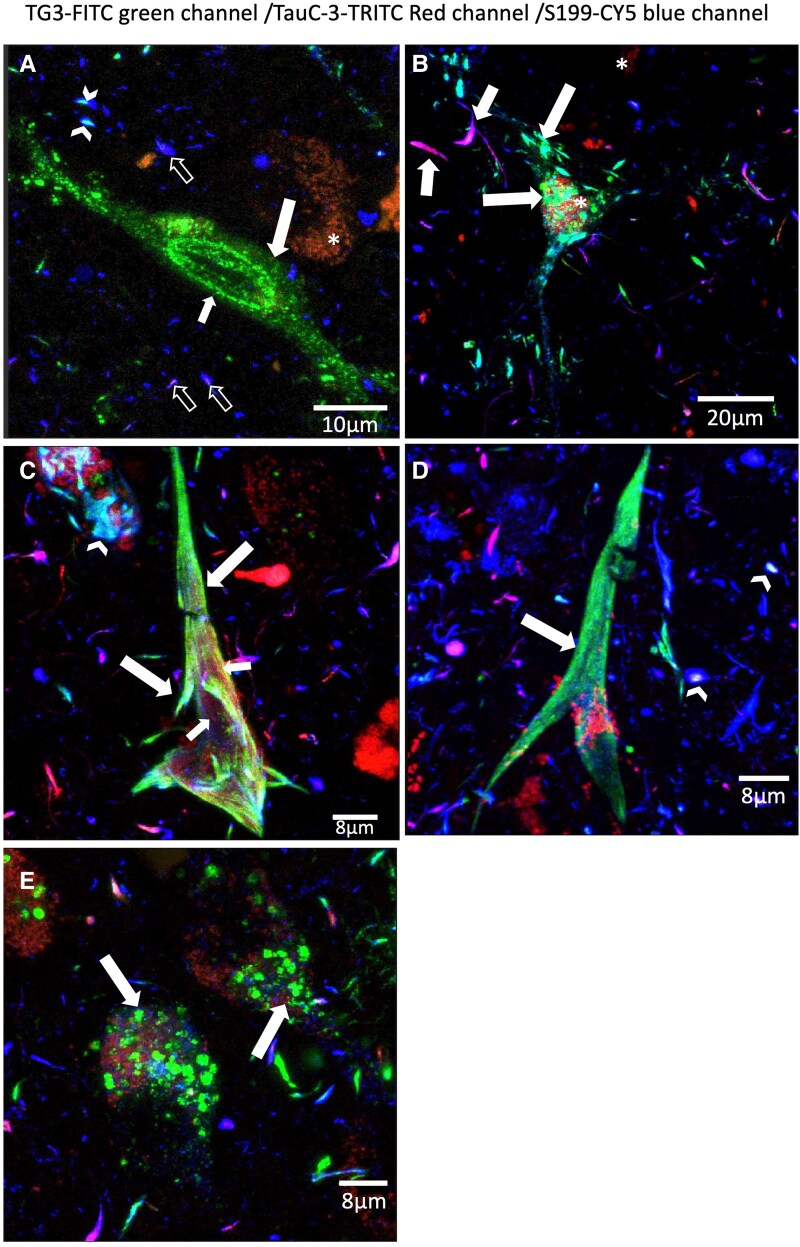
**Evaluation of tau protein with regional conformational change (TG3), truncated at Asp421 (TauC3) and phosphorylated tau at S199.** (**A**) Pre-NFT where colocalization of TG3 and S199 is evident. TG3 antibody immunoreactivity prevails. (**B**) Small tangles invade the soma of the neuron. In these small dense bundles of tau protein colocalize the three markers. (**C**) Intracellular NFT. Colocalization of TG3, TauC3 and S199 antibodies is observed, although the ratio of the three shows shades of colocalization (small and large arrows). In the vicinity, a NFT is observed where they colocalize to varying degrees (arrowhead). (**D**) Transitional NFT, TG3 antibody immunoreactivity prevails in relation to pS1998 antibody. (**E**) Granulovacuolar staining immunoreactive to TG3 (see Supplementary Fig. S1).

Triple immunostaining of TG3 (green channel), TauC-3 (red channel) and S199 (blue channel) antibodies showed different patterns of colocalization. Figure 2A shows diffuse granular staining that is predominantly positive for the TG3 antibody (green channel). Some small hints of S199 expression are observed in this cytoplasmic (large arrow) and perinuclear area (small arrow) granular aggregation. In the vicinity of this neurite, predominantly S199 antibody expression is observed (blue channel). TauC-3 and S199 immunoreactivity is observed in some neurites (Fig. 2A, empty arrow). In another neurite population, the three antibodies are colocalizing (Fig. 2A, arrowhead). The beed-like structures show high immunoreactivity to the three TG3-TauC-3-S199 markers (Fig. 2B, arrows); structures that invade the body of the neuron, in the periphery, dystrophic neurites are observed with a predominance of TauC-3 antibody expression (red channel) and colocalizing with S199 (blue channel) and slightly with TG3 (green channel; Fig. 2B, short arrows). The presence of TG3 and S199 immunoreactivity colocalizes in the majority of neuronal soma (Fig. 2C, arrow); in the middle portion of the neuronal soma the presence of TauC-3 antibody is predominantly observed (Fig. 2C, red channel, small arrows). In another NFT population TG3-TauC-3-S199 are colocalizing (Fig. 2C, arrowhead). At the periphery of this NFT, another NFT is observed, where the three antibodies colocalize (Fig. 2C, arrowhead). In the vicinity, dystrophic neurites are observed where S199 immunoreactivity predominates (blue channel). In some population of these neurites colocalizes with TauC-3 (Fig. 2C, Fuchsia colour). In Fig. 2D, a predominantly NFT is observed which was only evidenced by the TG3 antibody (Arrow). In the vicinity, dystrophic neurites are observed mainly positive for S199 and others colocalize with the TauC-3 antibody, while in another population of neurites all three markers colocalize (arrowhead). Additionally, granulovacuolar staining was commonly observed (Fig. 2E, arrow). These structures were mainly evidenced by the TG3 antibody. In the vicinity, dystrophic neurites are observed where the three antibodies directed against the tau protein have a differential colocalization pattern, see in Supplementary material Fig. S1.

### Triple immunostaining with antibodies directed against N- and C-terminal protein

**Figure 3:**
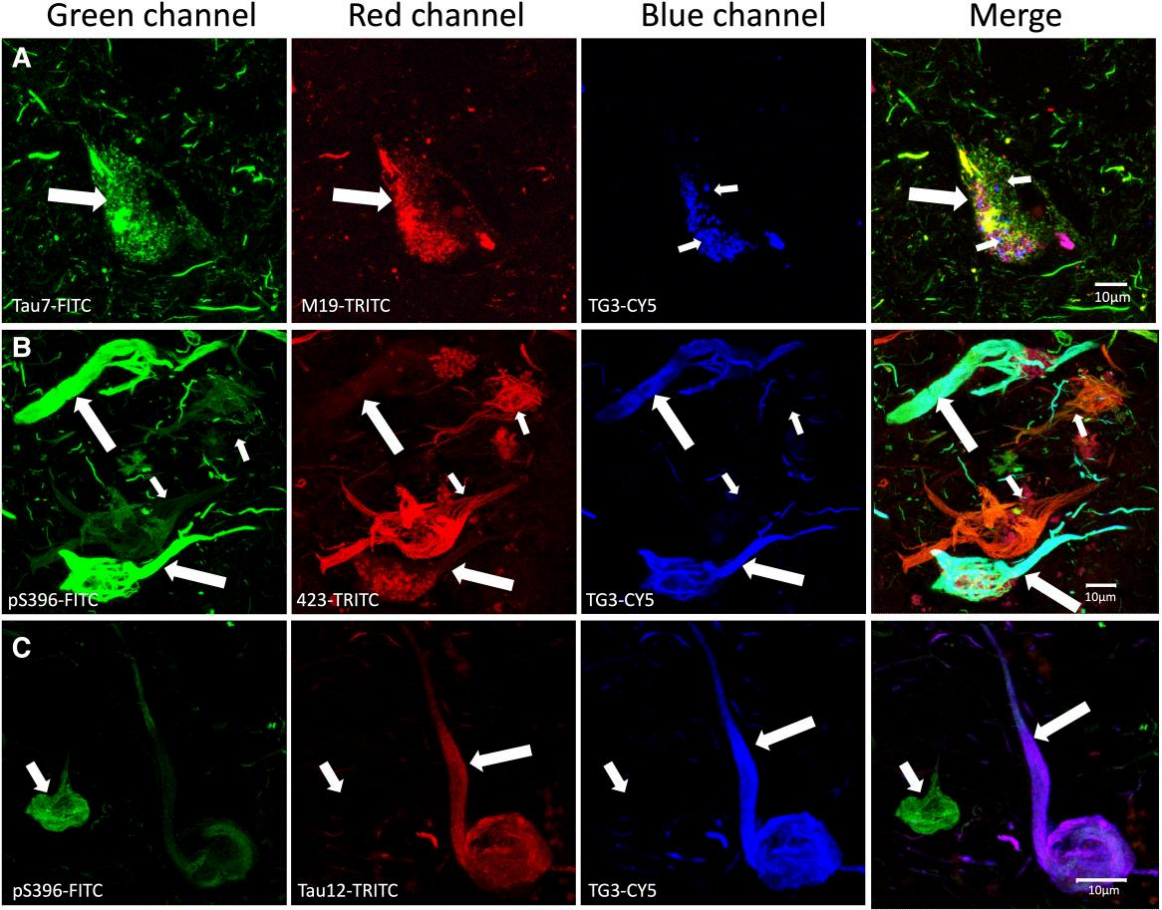
**Triple immunostaining of intact protein (tau7, M19 and tau12), truncated tau (423) and regional conformational change (TG3).** (**A)** NFT immunoreactive to intact tau markers in its carboxyl-terminal (tau7) and amino-terminal (M19) portions. However, TG3 antibody immunoreactivity evidenced preferential granulovacuolar staining. (**B)** NFTs of entorhinal cortex II evidenced an independence of TG3 and 423 antibody immunoreactivity. However, the highest colocalization was observed between pS396 and 423 antibodies and between pS396 and TG3. 423 does not colocalize with TG3. (**C)** TG3 immunoreactivity colocalized with tau12 (red channel, TRITC), which recognizes the first amino acids of the N-terminal portion (6–18 aa, large arrow). pS396 immunoreactivity did not colocalize with TG3 and tau12 (small arrow).

Diffuse granular staining was observed (Fig. 3A), which showed a high affinity for tau immunoreactive to Tau7 antibody (green channel, arrow) and M19 antibody (red channel), whereas tau protein immunoreactive to TG3 antibody showed a granular vacuolar staining (small arrow, blue channel), which did not colocalize with Tau7 and M19. The panel in Fig. 3B, shows triple staining with the antibodies pS396 (phosphorylation at S396; green channel), 423 (Truncation at E391; red channel) and TG3 (regional conformational change; blue channel). The group of NFTs have differential patterns of colocalization. In some of these NFTs, all three markers colocalize (Fig. 3B, large arrow); another population of NFTs is characterized by the absence of TG3 immunoreactivity (Fig. 3B, small arrows). Figure 3C shows an NFT in which TG3 antibody (blue channel) and Tau12 antibody (red channel) predominantly colocalize, whereas pS396 antibody immunoreactivity was very slight (green channel). Another NFT population was characterized by exclusively S396 immunoreactivity (Fig. 3C, Green channel, small arrow).

### Expression of epitopes against tau protein in NFTs

**Figure 4:**
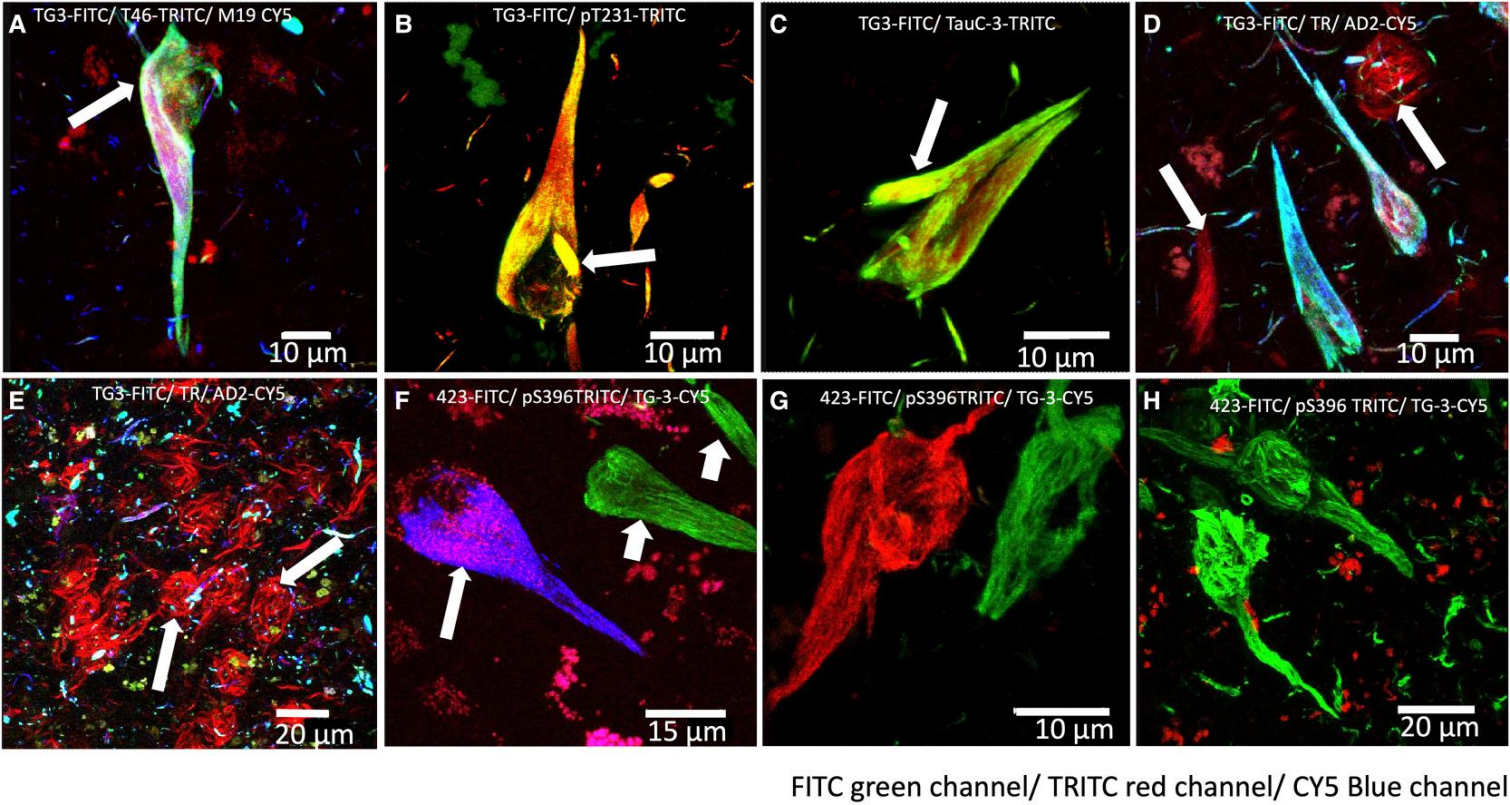
**Processing of tau protein in conformationally modified NFTs (TG3).** (**A)** TG3-T46 (C-terminal)-M19 (N-terminal) antibodies colocalized abundantly in the intracellular NFTs. (**B)** TG3 double staining with pT231. Both antibodies colocalize to varying degrees, with expression of phosphorylated tau protein in the T231 portion prevailing. (**C)** TauC-3 (asp421-truncated tau) antibody immunoreactivity colocalizes abundantly with TG3 antibody, with TauC-3 immunoreactivity prevailing. (**D)** Intracellular NFTs immunoreactive to TG3 colocalize with AD2 antibody and have affinity for the dye thiazine red. In the vicinity, two NFTs of lax fibrillar appearance are observed (arrows), which did not show immunoreactivity to TG3 and AD2. (**E)** NFTs of a lacuna of Layer II of the entorhinal cortex mainly evidenced by thiazine red dye (arrows). In the vicinity, dystrophic neurites are observed dystrophic neurites affine to thiazine red and immunoreactive to TG3 and AD2 antibody. (**F)** A population of NFTs evidenced colocalization between TG3 (blue channel) and pS396 (blue channel). Other NFTs with less compact appearance were only evidenced by antibody 423 (blue channel). (**G, H)** Neurofibrillar tangles with a lax appearance are affinity for antibody 423 (green channel) and another population of neurofibrillar tangles are affinity for antibody pS396 (red channel). TG3 antibody immunoreactivity was only observed in dystrophic neurites in the vicinity of these structures (see Supplementary Fig. S2).

Expression of intact N- (M19, blue channel) and C- (T46, red channel) antibodies colocalize abundantly with TG3 antibody (green channel, Fig. 4A, arrow). The immunoreactivity of the TG3 antibody with pS231 (Fig. 4B, arrow) colocalizes abundantly some areas; however, the immunoreactivity of antibody pT231 predominates in this NFT, as does the immunoreactivity of the TG3 antibody with TauC-3 (Fig. 4C, arrow). The Fig. 4D shows double immunostaining with TG3 (green channel) and AD2 (blue channel) antibodies, which colocalize abundantly. However, counterstaining with the thiazine red dye revealed a population of NFTs exclusively related to this dye (Fig. 4D, arrows). In the entorhinal cortex Layer II (Fig. 4E), a large number of NFTs with a lax appearance were observed, affine only to the thiazine red dye (arrows), and the antibodies colocalize to varying degrees in the dystrophic neurites encountered in the vicinity. The immunoreactivity of the TG3 antibody colocalizes with the pS396 antibody (Fig. 4F, large arrow). In other, NFTs in the vicinity were only positive to antibody 423 (Fig. 4F, small arrow). In Fig. 4G, immunoreactivity was not observed for TG3 antibody, but immunoreactivity was observed for antibody 423 (green channel) and antibody pS396 (red channel). In Fig. 4H, a lax-appearing NFT was observed, only immunoreactive to antibody 423 (green channel), see in supplementary Fig. 2.

### Immunostaining of the entorhinal Cortex II

**Figure 5:**
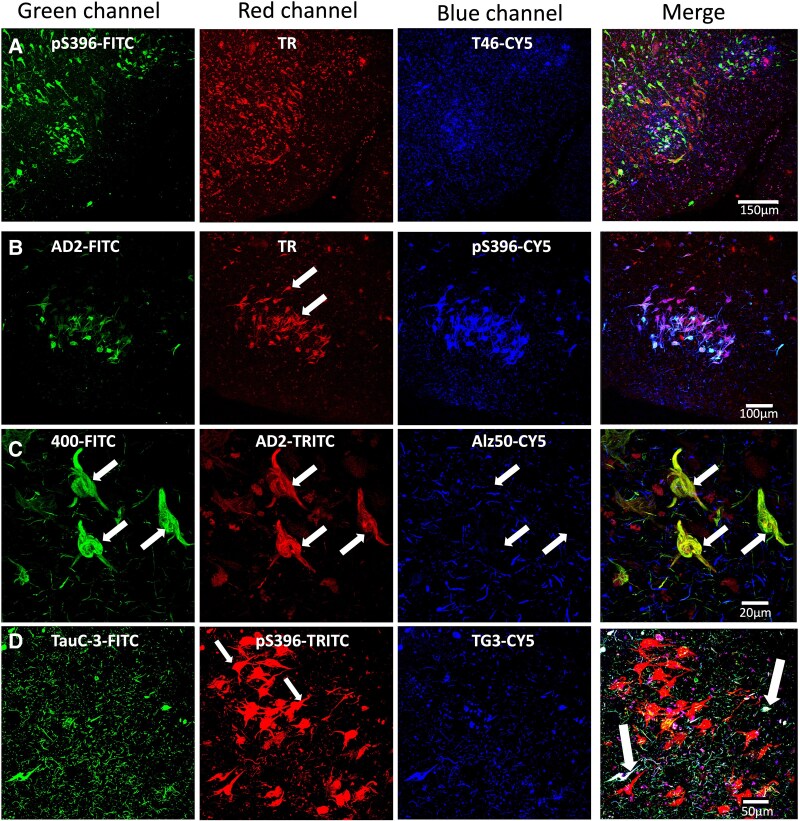
**Triple immunostaining in the population of NFTs in the lacunae of the entorhinal cortex Layer II.** (**A)** Double immunostaining with antibodies pS396 (green channel) and T46 (blue channel) counterstained with thiazine red dye (red channel). The pS396 antibody evidenced a significant population of NFTs, some colocalizing with the thiazine red dye, while the immunoreactivity of the T46 antibody was observed preferentially in dystrophic neurites, with few NFTs colocalizing with the three markers. (**B)** The population of MNF immunoreactive to the AD2 antibody (green channel) colocalizes with the pS396 antibody (blue channel) and the thiazine red dye (red channel), whereas other NFTs were only colocalized with the thiazine red dye. (**C)** NFTs immunoreactive to pS400 antibody (green channel) and AD2 (red channel) colocalized abundantly, Alz50 antibody (blue channel) immunoreactivity was observed only in dystrophic neurites. (**D)** Most of the NFTs are evidenced by the pS396 antibody (red channel) which did not colocalize with the immunoreactivity of the TauC-3 (green channel) and TG3 (blue channel) antibodies. Few NFTs had affinity for all three antibodies (arrow). TauC-3 and TG3 colocalized abundantly in dystrophic neurites.

**Figure 6:**
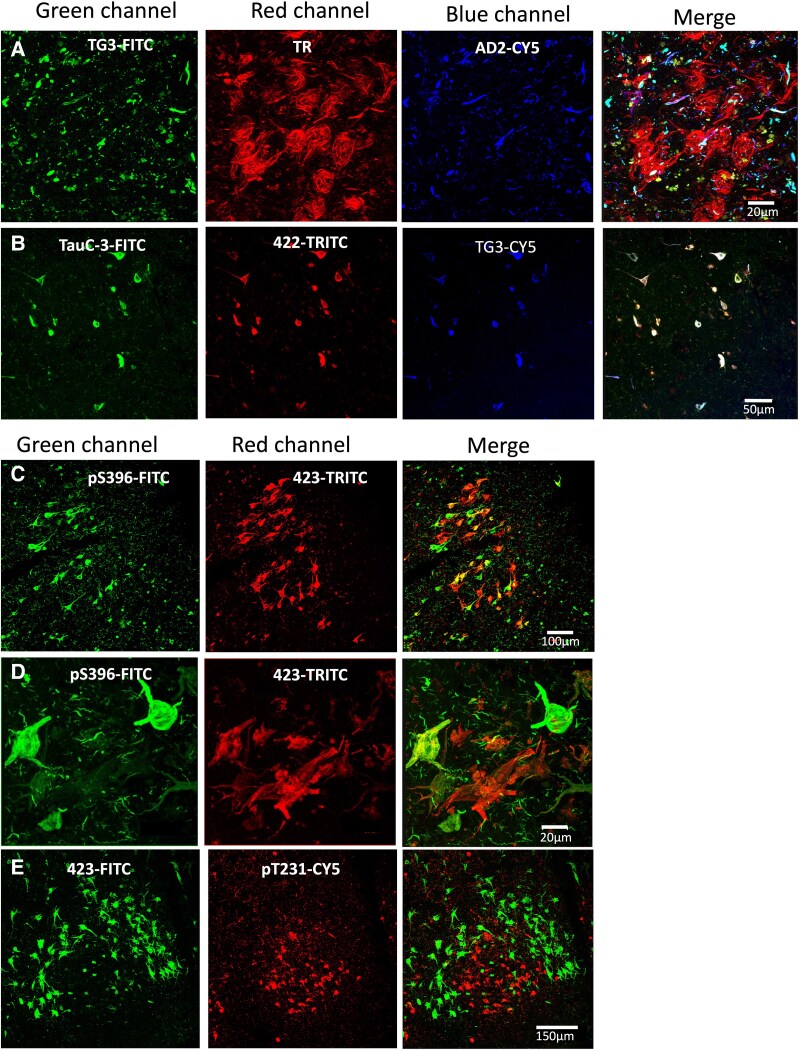
**Immunostaining in NFTs in the entorhinal cortex Layer II.** (**A)** The population of NFTs with affinity for the thiazine red dye (red channel) were not recognized by TG3 (green channel) and AD2 (blue channel) antibodies. (**B)** A population of NFTs showed colocalization with the three markers TauC-3 (green channel), pS422 (red channel) and TG3 (blue channel). (**C)** NFTs in the lacunae are abundantly evidenced by the two markers pS391 (green channel) and 423 (red channel). Some NFTs express only the 423 antibody (red channel). (**D)** In an amplification, the degree of colocalization between the two markers can be observed. (**E)** Immuroreactivity of antibody 423 (green channel) was occasionally observed bordering a gap of NFTs positive exclusively for pT231 antibody.

NFTs observed in entorhinal Cortex II (ERC II) are distributed in small lacunae. In AD, these lacunae showed a high affinity for the thiazine red dye (Fig. 5A, red channel), most of which colocalized with the PS396 antibody (Fig. 5A, green channel). Immunoreactivity of the T46.1 antibody recognizing the C-terminal portion only evidenced dystrophic neurites (Fig. 5A, blue channel). Some NFTs only showed affinity for the thiazine red (Fig. 5B, red channel, arrow). Immunoreactivity of the AD2 antibody (Fig. 5B, green channel) showed mainly colocalization with the pS396 antibody (Fig. 5B, blue channel), and the thiazine red (Fig. 5B, red channel). However, most of the pS396 antibody-positive NFTs colocalize abundantly with the thiazine red (Fig. 5B, merge). PS400 and AD2 antibody immunoreactivity colocalize abundantly in the NFTs (Fig. 5C, green and red channel, respectively, arrows) In the vicinity, dystrophic neurites immunoreactive to Alz50 antibody are observed (Fig. 5C, blue channel), which colocalize to varying degrees with antibody 400 and AD2. The truncated species evidenced with the TauC-3 antibody (Fig. 5D, green channel) and the antibody recognizing the regional TG3 conformational change showed very limited colocalization in NFT in the Layer II of the entorhinal cortex, where they were mainly strongly evidenced by the thiazine red (Fig. 5D, arrows). Colocalization with all three markers was very limited in the population of these neurons (Fig. 5D, merge, large arrow). TG3 and AD2 antibody immunoreactivity (Fig. 6A, green and blue channel) colocalize mainly in the population of neurites associated to varying degrees with the thiazine red. NFTs from this entorhinal cortex area were only affine to the thiazine red (Fig. 6A, red channel). TauC-3 (Fig. 6B, green channel), pS422 (Fig. 6B, red channel) and TG3 (Fig. 6B, blue channel) evidenced a low number of NFTs in Layer II entorhinal cortex. However, in the observed NFTs all three markers showed colocalization (Fig. 6B, merge channel). In Fig. 6C and D, immunoreactivity of pS396 (green channel) and 423 antibody (red channel) showed independent NFT populations and another colocalizing population, with NFTs immunoreactive to 423 being more abundant. Dystrophic neurites immunoreactive to pS396 antibody are abundant in the vicinity of these lacunae of NFT. Immunoreactivity to the 423 antibodies and pT231 showed an abundance of NFTs in these lacunae, with complete independence between the two markers. Positive NFTs were observed for antibody 423 (Fig. 6E, green channel) and pT231 (Fig. 6E, red channel).

### Distribution of the number of TG3 antibody-positive NFTs across Braak stages

**Figure 7:**
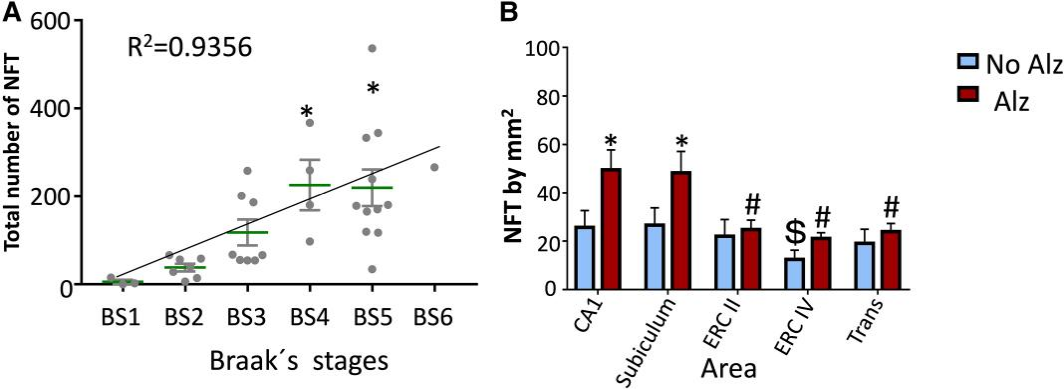
**Number of lesions immunoreactive to TG3 antibody in Alzheimer and control cases.** (**A**) Density of TG3 antibody-immunoreactive NFTs in control and Alzheimer’s cases. The number of NFT by mm^2^ increased in correlation with the Braak stage (R^2^ = 0.9649), the NFTs in Braak stages 5 (diff of ranks = 104; q = 5.283) and 4 (diff of ranks = 81; q = 4.115) were significantly more abundant compared to Braak stage 1 (*P* = 0.001). Braak’s stage I *n* = 3, Braak’s stage II *n* = 7, Braak’s stage III *n* = 13, Braak’s stage IV *n* = 7, Braak’s stage V *n* = 9 and Braak’s stage VI *n* = 1. (**B**) The number of lesions per area in the hippocampus and entorhinal cortex. In control cases, the number of lesions in all areas was very homogeneous. However, in Alzheimer cases, a higher number of lesions were observed in the CA1 and Subiculum area while the number of lesions remained constant in the entorhinal Cortex II, IV and IV. Each bar represents the media ± SEM. * P < 0.05 ALZ (Alzheimer cases) versus NO–ALZ (controls); # *P* < 0.05 within ALZ CA1 and Subiculum versus ERC II, ERC IV and Transentorhinal cortex. $ *P* < 0.05 Within No—ALZ cases CA1 and subiculum versus ERC IV. Two-way repeated measures (ANOVA) (one factor repetition) was applied, and the Student—Newman—Keuls *post hoc* test was used for multiple comparisons; AD *n* = 15; no AD *n* = 25.

The CPLL cases are part of an encephalon collection characterized by Braak stages, APOE4 and degree of cognitive impairment. The number of TG3 antibody-positive lesions in Stages BS1 and BS2 showed limited abundance. In later stages, BS3, BS4 and BS5 and BS6 an increase in the number of lesions immunoreactive to the TG3 marker was evident (Fig. 7A).

### Density of TG3 antibody-immunoreactive NFTs in control and Alzheimer's cases

The number of NFT by mm^2^ increased in correlation with the Braak stage (R2 = 0.9649), the NFTs in Braak stage 5 (diff of ranks = 104; q = 5.283) and 4 (diff of ranks = 81; q = 4.115) were significantly more abundant compared to Braak stage 1 (*P* = 0.001) (Fig. 7A). The number of TG3 antibody-positive NFTs in each of the areas analysed was always higher in AD compared to control cases, *F*(4) = 3.761; *P* = (0.006) . In the CA1, *P* = (0.017), and subiculum area *P* = (0.005), 120–130 NFTs/mm^2^ were found in AD compared control cases, while in the transentorhinal cortex and entorhinal cortex Layer II was between 60 and 70 TFNs/mm^2^). NFT was found in lower proportion in Layer IV entorhinal cortex (50 NFT/mm^2^. There were no significant differences between transentorhinal and entorhinal Cortex II and IV (*P* = 0.579; *P* = 0.643 and *P* = 0.318, respectively) (Fig. 7B).

### TG3 antibody-immunoreactive NFTs associated with Braak stages by area

**Figure 8:**
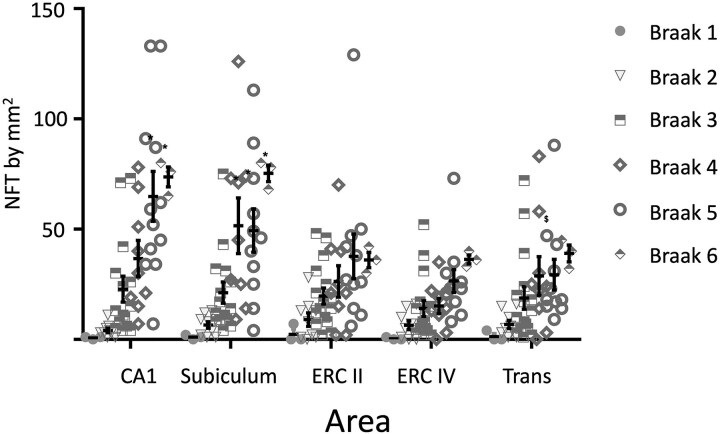
**Number of lesions evidenced by TG3 antibody per area in the hippocampus, entorhinal and transentorhinal cortex**. TG3 antibody immunoreactivity was observed with a higher abundance in Braak stages I and II. In Stages III and IV, they remain constant and increase in all areas. In Stages V and VI, an increase is observed in CA1 and Subiculum areas while the number of lesions decreases in the entorhinal cortex II. Individual data is represented, as well as the media ± SEM. **#**  *P* < 0.05 in Braak’s stage IV when were compared ERC IV versus Subiculum; **$**  *P* < 0.05 in Braak's stage V when we were compared CA1 versus ERC II, Transentorhinal cortex and ERC IV; * *P* < 0.05 when are compared within the areas CA1 (Braak V and Braak VI versus Braak I, Braak II, Braak III and Braak IV) and Subiculum (Braak IV, Braak V and Braak VI versus Braak I, Braak II and Braak III). Two-way analysis of variance were performed and the Student—Newman—Keuls *post hoc* test was used for multiple comparisons. Braak’s stage I *n* = 3; Braak’s stage II *n* = 7; Braak’s stage III = 13, Braak’s stage IV = 7; Braak’s stage V = 9; Braak’s stage VI = 1.

The number of NFTs throughout the process of AD evolution characterized by TG3 antibody showed a considerable increase according to the severity of the disease, *F*(5) = 24.509; (*P* = 0.001) (Fig. 8, Braak stages I-VI). The number of NFTs in Braak stage I was very limited in hippocampal areas. The most abundant expression was observed in the transentorhinal layer and Layer II of the entorhinal cortex (*P* = 0.667). The affinity of TG3 antibody in Layer IV of the entorhinal cortex was minimal (Fig. 8). In Braak stage II, a very homogeneous number of NFT/mm^2^ was observed in all areas quantified, being in the transentorhinal cortex more abundant, no differences were found between the areas. In Braak stage III, an increase in the number of NFTs was observed in all areas, predominantly in the CA1 area. However, there were differences between the areas (*P* = 0.775 CA1 versus entorhinal cortex Layer IV). The rest of the areas remained the same between 40 and 45 NFT/mm^2^. A slight decrease in the number of NFTs immunoreactive to TG3 antibody was observed in the entorhinal cortex Layer IV (35 NFTs/mm^2^). A higher number of TG3 antibody-positive NFTs was observed in Braak stage IV. In entorhinal Cortex IV, there was a lower number of NFTs/mm^2^ than subiculum (*P* = 0.002). In Braak stage V, CA1 had more NTFs/mm^2^ than entorhinal cortex Layer II (*P* = 0.06); transentorhinal cortex (*P* < 0.001) and entorhinal cortex Layer IV. Braak's stage VI, a higher abundance of NFTs was observed in area CA1 (*P* < 0.001 versus Braak I, Braak II and Braak III and *P* = 0.023 versus Braak IV) and subiculum (*P* < 0.001 versus Braak I, Braak II and Braak III). However, in Braak’ stage VI, the number of NFTs in the entorhinal cortex was lower, remaining in greater proportion in CA1 and subiculum. CA1 also presented more NFTs/mm^2^ in Braak's stage V than Braak I, Braak II, Braak III (*P* < 0.001) and Braak IV (*P* = 0.003). Subiculum was also higher in Braak's stage V (*P* < 0.001 versus Braak I, Braak II and Braak III) and Braak's stage IV (*P* < 0.001 versus Braak II and *P* = 0.002 versus Braak III).

### Clinical severity and presence of NFT immunoreactive to TG3 and AT8

**Figure 9:**
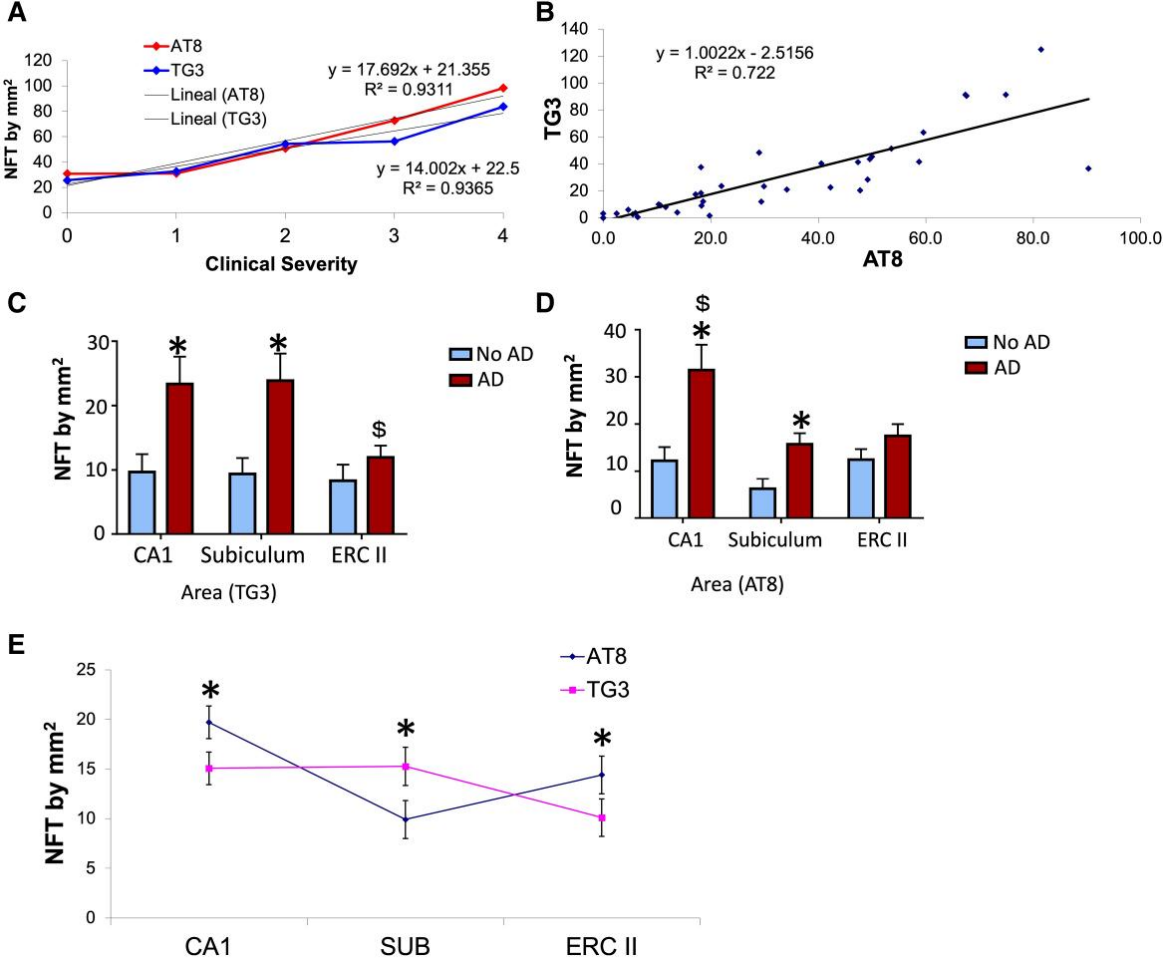
**Number of lesions evidenced by TG3 and AT8 antibodies and clinical severity. (A)** The graph shows an increase in the number of lesions related to the two phosphorylated tau markers as the clinical severity of the patient increases. (**B)** The number of lesions for AT8 and TG3 tend to show an increasing trend in the number of lesions related to these markers. (**C, D)** The number of lesions for both markers behave the same. (**C**) NFT by mm^2^ by area labelled by AT8, each bar represents the media ± SEM (* *P* < 0.005 Ad versus control; and **$**  *P* < 0.005 CA1 and Subiculum versus ERC II), no AD *n* = 25; AD *n* = 15. (**D)** NFT by mm^2^ by area labelled by TG3, each bar represents the media ± SEM. (* *P* < 0.005 Ad versus control; and **$**  *P* < 0.005 CA1 versus Subiculum and ERC II) Two-way analysis of variance were performed for both graphs and the Tukey test for multiple comparisons. (**E)** Both markers have a tendency to decrease in the entorhinal cortex and remain in the CA1 area. However, TG3 antibody was more abundant in the subiculum. Each point represents the media ± SEM (* *P* < 0.05, AT8 versus TG3 compared by area). Paired samples *t*-test.

The presence of NFTs showed an increase as clinical severity progressed. The stages showing a significant difference were between Stages 0, 3 and 4 clinical severity (Fig. 9). This behaviour was similar when comparing the number of lesions positive for TG3 and AT8 antibody. The two antibodies tend to increase as clinical severity increases. It starts at Stage 0 with 22 NFT/mm^2^ (TG3) and 30 NFT/mm^2^ (AT8) and culminates at Stage 4 with 90 and 100 NFT/mm^2^. For NFT versus severity with AT8 (R^2^ = 0.9311; y = 17.692 × + 21.355) and for NFT versus severity with TG3 (R^2^= 0.9365; y = 14.002 × + 22.5) (Fig. 8).

The scatter analysis shows a similar trend in the increase of lesions positive for TG3 and AT8 markers (R^2^ = 0.722; y = 1.0022 × —2.5156) (Fig. 9B). The total number of lesions immunoreactive to TG3 antibody (Fig. 9C) and AT8 (Fig. 9D) in cases with AD was higher compared to control cases. For TG3, CA1 and subiculum were higher in AD versus control (*F*(1) = 17.998; *P* < 0.01), but also within the AD cases, the NFT in CA1 and Subiculum were more abundant than ERC II (*P* = 0.042 and *P* = 0.031, respectively). AT8 was similar to TG3, with AT8, the NFT were more abundant in CA1 and subiculum in AD compared to control (*F*(1) = 21.285; *P* = 0.001), and when were compared the areas in AD cases, CA1 has more NFT versus Subiculum and ERC II (*P* = 0.003 and *P* = 0.010, respectively). The number of NFTs immunoreactive to TG3 antibody and AT8 is mostly present in the CA1 and subiculum compared with the entorhinal cortex Layer II. In CA1, AT8 was higher than TG3 (t = 2.822; df = 35; *P* = 0.008), in Subiculum TG3 was higher than AT8 (t = −2.797; df = 35; *P* = 0.008), and for ERC II, AT8 was higher than TG3 (t = 2.271; df = 35; *P* = 0.029) (Fig. 9E).

### Number of TG3-positive NFTs and presence of ApoE genotype

**Figure 10:**
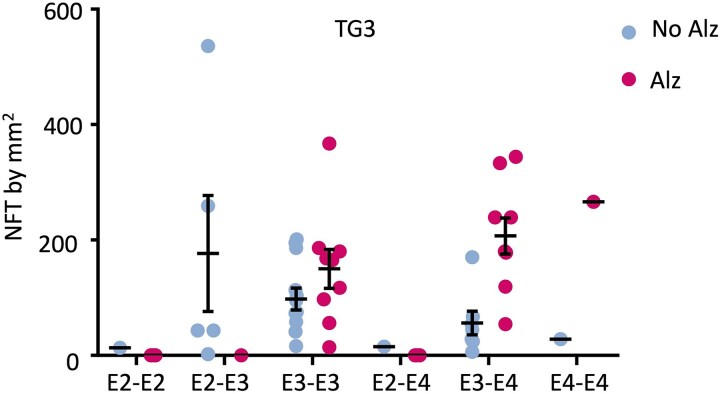
**Number of lesions in Alzheimer's disease and control cases in ApoE genotyping.** A higher number of lesions is observed in ApoE4 homozygosity. The number of lesions is lower in cases with a combination of ApoE3 and ApoE4 and decreases in ApoE3 homozygotes. In control cases, the presence of NFTs was mostly observed in ApoE2 and ApoE3 cases. Individual data are represented, as well the media ± SEM. Two-way analysis of variance were performed. E2-E2 *n* = 1; E2-E3 *n* = 4; E2-E4 *n* = 1; E3-E3 *n* = 19; E3-E4 *n* = 13; E4-E4 *n* = 2.

CPLL cases were genotyped for ApoE. The highest number of lesions was present in Alzheimer cases with ApoE4 homozygous genotyping, followed by the mixed (ApoE3-ApoE4) with a lower number of lesions immunoreactive to TG3 antibody compared to control cases (Fig. 10). In the control cases, the presence of ApoE2 and ApoE3 showed a higher abundance of lesions than the other mixed cases of ApoE genotyping. However, despite this tendence, statistically there was no significant difference: *F*(5) = 0.880, *P* = 0.506 for ApoE and *F*(1) = 0.516, *P* = 0.478 for AD versus control cases.

## Discussion

### Expression pattern of tau and TG3

Post-translational changes of tau can modify its structure and conformation.^15,36-38^ The addition of phosphates to the constituent amino acids of the tau protein can favour the regional folding of the molecule, favouring intramolecular binding.^25,39^ In our study, we have been able to analyse a conformational change evidenced by the TG3 antibody, which evidences the phosphorylation-dependent intramolecular relationship of amino acids 231 and 235.^24,40^ Expression of the TG3 antibody evidenced a sequence of tau protein aggregation in the neuronal soma. The first aggregation of tau protein is characterized by a diffuse granular appearance, where tau protein is not associated with fibrillar structures (pre-NFT). Furthermore, these structures have no affinity for the red dye thiazine (red fluorescent dye that has affinity for tau protein and amyloid-beta peptide, when assembled into filaments with the beta-folded conformation).^15,16,35^

Subsequently, the small tangles were observed, which are already affine to the thiazine red dye. These small tangles tend to fuse and elongate invading the body of the neuron, giving way to the next stage, which is the intracellular NFT.^15,16,35,41^ TG3 antibody immunoreactivity evidenced a population of transitional neurons but did not evidence extracellular NFT. These results suggest that TG3 antibody immunoreactivity is very stable and practically follows the evolution of NFT formation. Thus, TG3 could be an excellent biomarker in neurodegenerative evolution in AD.

### Processing of tau and TG3

The processing of tau associated with the phosphorylation mechanism is more likely to be able to evidence a sequence of events associated with phosphorylation in pre-TNFs.^15^ This is because the changes are very subtle, and the processing of phosphorylation events are closely related. In previous studies from our laboratory, we have suggested that prior to the regional conformational change evidenced by the TG3 antibody in tau, the amino acid Thr231 and subsequently Ser235 must be phosphorylated. This favours the regional conformational change evidenced by TG3. Subsequently, it is phosphorylated at amino acid 202–205 (AT8 antibody), to give way to a third phosphorylation 212–214, giving way to a second regional conformational change evidenced by the AT100 antibody.^25,39^ The set of these regional conformational changes (TG3, AT100) favour the structural conformational change evidenced by the Alz50 antibody.^22,23^ It has also been observed that since the presence of phosphorylated tau in Asp421 (evidenced by the TauC-3 antibody),^19,37^ an action carried out by caspase 3. Truncation of tau favours its polymerization.^10,42^ The group of Binder L. evidenced a sequence of events in the well-formed NFT, suggesting that the first conformational change is recognized by Alz50 (N-terminal portion intact; C-terminal may or may not be intact). It then continues with truncation at amino acid 421 (Tau-C3) to give way to another conformational change Tau66,^43^ which does not require its N-terminal portion to be intact. However, this study evidence very separate events in the processing and does not consider the processing of phosphorylated tau. In our study we were able to observe that the TG3 antibody evidenced pre-NFTs, (small intracellular NFT) where it was associated with intact tau protein in its N- and C-terminal intact portion. It was also observed that the C-terminal portion is very sensitive to proteolysis as evidenced by Tau-C3 and the loss of immunoreactivity of Tau7. The phosphorylation of the C-terminal portion (evidenced by the pS396 and AD2 antibodies) is expressed as soon as the tau protein has been added in the small NFTs up to the transitional tangles. In all these events, TG3 is present colocalizing with these markers. However, none of these markers have affinity for the NFTs that are evidenced by the thiazine red dye and the antibody 423.^11,12,33^ Antibody 423, which recognizes truncation at Glu391, is abundantly found in extracellular NFTs. However, the epitope evidenced by antibody 423 could be observed from preNFTs (unpublished results) and occurs during all tau aggregation events in the neuronal soma. Studies by Fasulo 2002 evidenced that overexpression of Glu391-truncated tau protein in cell culture cells died by apoptosis.^44^ In AD, few NFTs have been associated with death by apoptosis. Therefore, neuronal death in AD is carried out by massive accumulation of tau PHFs. It has been suggested that the tau molecules in PHFs have an antiparallel arrangement.^45^ These results suggest that the fragment (92–95 aa) truncated in Glu391 could be being concealed by conformational changes of the molecule in its N-terminal portion, thus evading the neuron that the presence of this fragment would result in a rapid and accelerated apoptosis process.^44^ The Braak stage classification is based on the presence of NFTs in the hippocampus. These lesions follow a specific pattern associated with the perforant pathway.^46^ The number of TG3-positive NFTs in the different Braak stages increases over the stages; however, they do not show a considerable difference. This corroborates what was observed in the aggregation patterns of tau immunoreactive to the TG3 antibody; it reaches a transitional point of intracellular NFT, but does not evidence extracellular NFT, which is specifically constituted by the PHFcore. This fragment is recognized exclusively by antibody 423 (truncation at Glu391).^9,47^ Differentiating between control and AD cases and according to area, it can be observed that in the control cases the immunoreactivity of the TG3 antibody is homogeneous in all areas. In AD cases, it can be observed that there is a higher number of NFTs in the CA1 and subiculum while in the transentorhinal cortex and entorhinal II there is a lower number of lesions. This suggests that TG3 epitope is lost because it gives way to the extracellular 423 antibody-positive NFTs, which may be associated with the high sensitivity of these areas. The CA1 and subiculum area have been suggested to be areas more resistant to neurodegeneration. The decrease of NFTs in Layer IV of the entorhinal cortex is associated with the outflow of information from the hippocampus to the neocortex (perforant pathway).^48,49^

### Sensitivity of Layer II of the entorhinal cortex to neurodegeneration

The entorhinal cortex is an area involved in the cognitive function of memory formation. Braak and Braak stages evidenced a specific sequence of the presence of NFTs in the hippocampus and over time have been associated with cognitive impairment in the AD patient.^46^ Lesions in the entorhinal cortex are carried out in a lateral manner in the lacunae of Layer II of the entorhinal cortex. This lateral neurodegeneration transmission could be favoured by the communicating junctions that neurons develop in their dendritic tree. In the entorhinal Cortex II and IV and hippocampus (subiculum, CA1-CA4), an increase in the number of TG3 antibody-positive NFTs is observed. A greater number of lesions in the transentorhinal cortex can be seen in Stage I; in Stage 2, it increases homogeneously in all areas, prevailing in the transentorhinal cortex. In Stages 3 and 4 (where the first symptoms of the disease are expressed), the number of lesions increased considerably and homogeneously in all areas. In Stages 5 and 6, where clinically drastic cognitive changes occur, the number of lesions is higher in CA1 and subiculum. In Stage 6, a decrease in the number of lesions immunoreactive to TG3 antibody is observed in the entorhinal cortex Layer II, which is associated with molecular changes of tau protein, giving way to extracellular NFTs immunoreactive to antibody 423.

A higher number of lesions was observed in cases with homozygous genotyping of Apo4, which is associated as an important risk factor for the development of Alzheimer's disease. A lower proportion of the number of lesions was evidenced in ApoE3 homozygosis, which has been associated as a minor risk factor for the development of Alzheimer's disease. Interestingly, ApoE2 and ApoE4 heterozygosity and a considerable number of TG3 immunoreactive lesions were observed in the control cases. This observation should be considered in future studies in association with lifestyles that could be implicated in the development of Alzheimer's disease.

### Conclusions

1. TG3 is associated with early stages of neurodegeneration, favouring together with the AT100 antibody the structural conformational change evidenced by the Alz50 antibody.
2. The TG3 antibody epitope suggests being very stable in terms of proteolysis of tau in its N-terminal and C-terminal portion.
3. The entorhinal cortex evidenced to be a very sensitive and fast processing area in neurodegeneration.
4. Neurodegeneration of the entorhinal cortex Layer II initially takes place laterally in the lacunae of the entorhinal cortex, whereas the formation of tangles in subiculum, CA1-CA4 is slower.

## Supplementary Material

fcaf091_Supplementary_Data

## Data Availability

No new software and/or algorithms, in-house scripts or programmes were generated to support this study. Requests for the data sets used in the present study will be promptly reviewed by the corresponding authors and the Universidad Politécnica de Pachuca to verify whether the request is subject to any intellectual property or confidentiality obligations. Anonymized data can be shared by request from any qualified investigator for the sole purpose of replicating procedures and results presented in the article, provided that data transfer is in agreement with México legislation. Requests received will be reviewed by the Universidad Politécnica de Pachuca Committee to verify whether these are subject to any intellectual property or confidentiality obligations and compliance with ethical and data protection standards. All requests for code used for data analyses and data visualization will be promptly reviewed by the corresponding authors and the Universidad Politécnica de Pachuca.
