# Supplementary material for: Vulnerability of the entorhinal cortex II to neurodegeneration in Alzheimer’s disease
Source: Brain Commun. 2025 Feb 26;7(2):fcaf091. doi: 10.1093/braincomms/fcaf091 (PMC11897590; doi:10.1093/braincomms/fcaf091)
Supplement: fcaf091_Supplementary_Data [file fcaf091_supplementary_data.pdf]

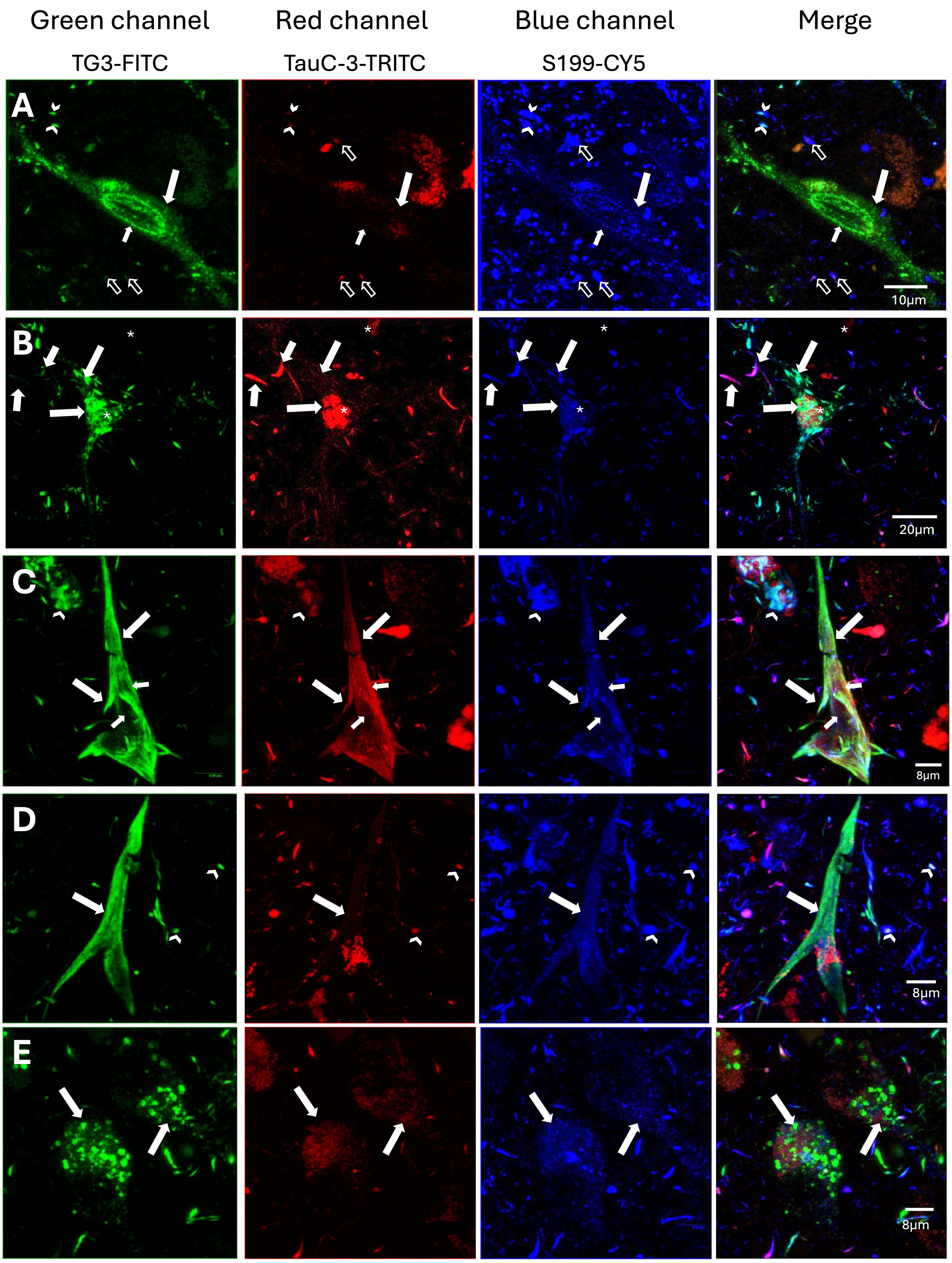

Supplementary Figure 1. Triple Immunolabelling Evaluation of tau protein with regional conformational change (TG3-FITC, green channel), truncated at Asp421 (TauC3-TRITC, Red channel) and phosphorylated tau at S199 (CY5, blue channel). A) Neurofibrillary Pretangle. Abundantly evidenced by the TG3 antibody in the green channel; showed a diffuse granular staining in the cytoplasm and dendrites (large arrow), with intense perinuclear staining (small arrows), which colocalizes with the immunoreactivity of the S199 antibody (blue channel). No immunoreactivity was observed with the TauC-3 antibody (red channel, large and small arrows); dystrophic dendrites are observed in the periphery, which showed affinity to all three antibodies (empty arrows and arrowheads). **B)** Immunoreactivity in small intraneuronal tangles immunoreactive to the TG3 antibody (FITC, green channel, large arrows) abundantly colocalizes with the S199 antibody (CY5, blue channel, large arrow). These structures colocalize to various degrees with the TauC-3 antibody (TRITC, red channel), with dystrophic neurites in the vicinity that colocalize to various degrees with all three antibodies (small arrows). **C)** Intracellular Neurofibrillary Tangle. TG3 antibody immunoreactivity abundantly colocalizes with TauC-3 and to a lesser extent with S199 (blue channel, large arrow). TauC-3 antibody expression is abundantly observed in certain areas of the neurofibrillary tangle (red channel, small arrow), which does not colocalize with TG3 and S199. **D)** Intracellular neurofibrillary tangle, immunoreactive to TG3 antibody (green channel, arrow) and S199 (blue channel, arrow). The TauC-3 antibody showed no affinity for this structure. Dystrophic neurites are observed in the periphery, which colocalize all three markers (arrowhead). **E)** Granulo-vacuolar staining exclusively immunoreactive with the TG3 antibody (green channel, arrows). The S199 antibody showed diffuse granular immunoreactivity in the neuronal soma (blue channel, arrows), while the TauC-3 antibody immunoreactivity did not show immunoreactivity for this granulo-vacuolar staining (red channel, arrows). Dystrophic dendrites are observed in the vicinity, which demonstrate various degrees of colocalization among the three tau markers.

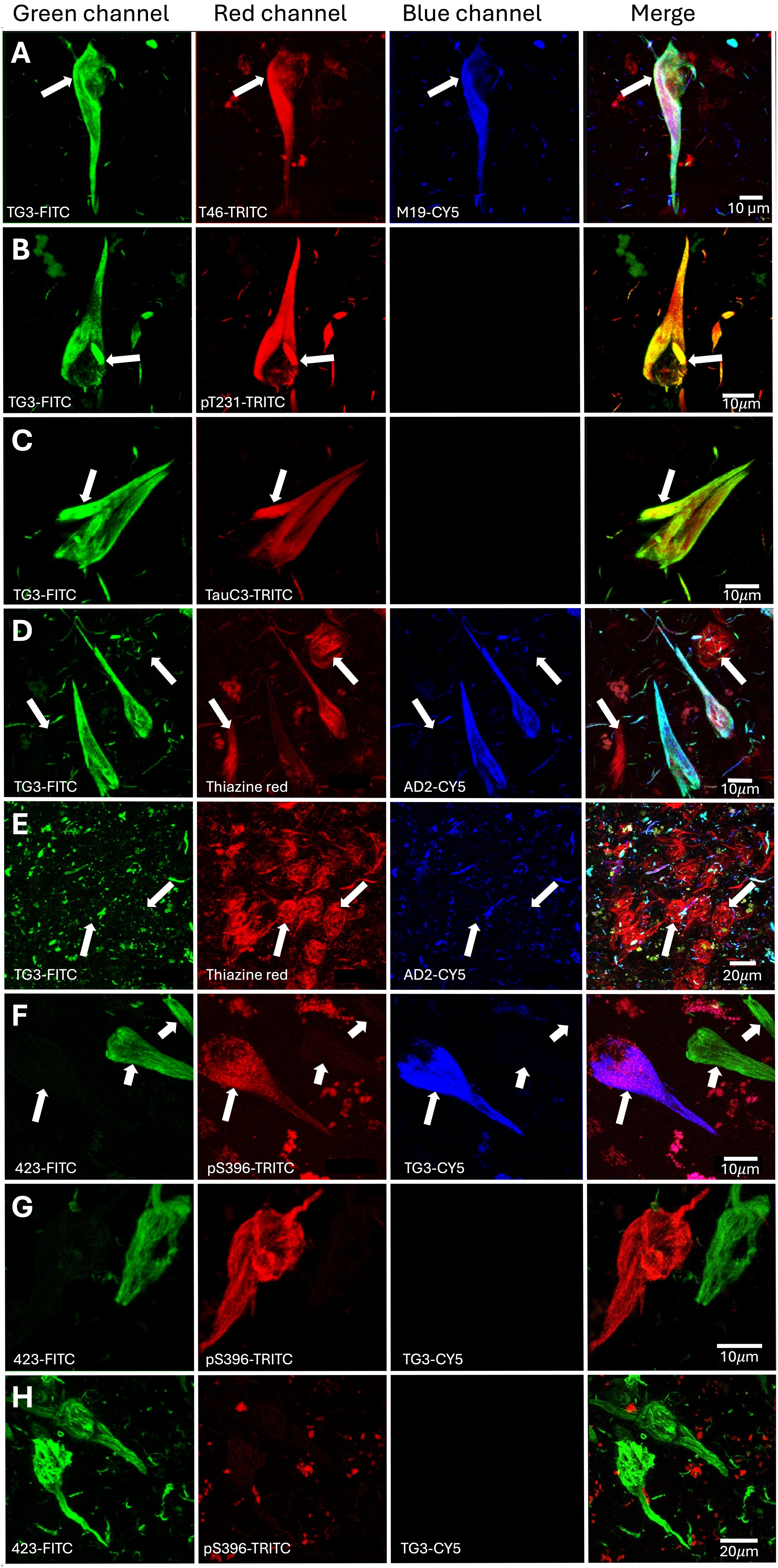

Supplementary Figure 2. Double and Triple Staining of Neurofibrillary Tangles with Intact Tau Protein Markers (T46, M19), Phosphorylated (TG3, pT231, AD2, 396) and Truncated (TauC-3, 423). A) Triple immunostaining with TG3 antibodies (green channel), T46 (red channel), M19 (blue channel) demonstrates colocalization of all three markers (arrow). **B)** Double immunostaining with TG3 antibody (green channel), which abundantly colocalizes with pT231 antibody (arrow). A middle portion of the neuron is primarily evidenced by the pT231 antibody (red channel). In the vicinity, dystrophic neurites with the same colocalization characteristics are observed. **C)** Double immunostaining of TG3 antibody (green channel) with TauC-3 antibody (red channel), both markers abundantly colocalize, with a portion of the neurofibrillary tangle showing unique TauC-3 antibody expression. **D)** Double immunostaining with TG3 (green channel) and AD2 (blue channel) antibodies, counterstained with thiazine red dye. Two neurofibrillary tangles immunoreactive to TG3 antibody (green channel) are observed, which abundantly colocalize with AD2 antibody (blue channel) and were evidenced by thiazine red dye (red channel). Two neurofibrillary tangles evidenced exclusively by thiazine red dye (red channel) with a loose fibrillar appearance (arrows). **E)** Group of neurofibrillary tangles from the entorhinal cortex layer II are evidenced only with thiazine red dye (red channel), with a loose fibrillar appearance. They showed no affinity for TG3 (green channel) and AD2 (blue channel) antibodies. Dystrophic neurites are observed in the periphery, which colocalize to various degrees with the three markers. **F)** Triple immunostaining with 423 antibody (green channel, short arrow) which does not colocalize with the immunoreactivity of 396 antibody (red channel, short arrow) and TG3 (blue channel). A neurofibrillary tangle immunoreactive for pS396 (red channel, large arrow) is observed, which abundantly colocalizes with TG3 antibody (blue channel, large arrow). **G)** Triple staining with 423 antibody (green channel) which showed no affinity for pS396 (red channel) and TG3 (blue channel) antibodies. Another neurofibrillary tangle was evidenced exclusively by the pS396 antibody (red channel). **H)** Neurofibrillary tangles with a loose fibrillar appearance immunoreactive to 423 antibody (green channel) did not colocalize with pS396 (red channel) and TG3 (blue channel) antibodies.
